# Non‐reproducible signals of adaptation to elevation between open and understorey microhabitats in snapdragon plants

**DOI:** 10.1111/jeb.13973

**Published:** 2021-12-20

**Authors:** Anaïs Gibert, Sara Marin, Pierick Mouginot, Juliette Archambeau, Morgane Illes, Gabriel Ollivier, Alice Gandara, Benoit Pujol

**Affiliations:** ^1^ PSL Université Paris EPHE‐UPVD‐CNRS USR 3278 CRIOBE Université de Perpignan Perpignan Cedex France; ^2^ Laboratoire Évolution et Diversité Biologique (EDB UMR 5174) Université Fédérale de Toulouse Midi‐Pyrénées, CNRS, IRD, UPS Toulouse France; ^3^ BIOGECO INRA University of Bordeaux Pessac France

**Keywords:** adaptation, *Antirrhinum majus*, common garden, elevation, germination, phenotypic plasticity, reproducibility, shade

## Abstract

Experimental studies on local adaptation rarely investigate how different environmental variables might modify signals of adaptation or maladaptation. In plant common garden experiments, signals of adaptation or maladaptation to elevation are usually investigated in open habitats under full light. However, most plants inhabit heterogeneous habitats where environmental conditions differ. Understorey microhabitats are common and differ in terms of tree shade, temperature, water availability, microbiota, allelochemicals etc. Germination is a fitness‐related trait of major importance for the adaptation of plants to contrasted climate conditions. It is affected by shade in snapdragon plants (*Antirrhinum majus*) and many other plant species. Here, we tested for the reproducibility of signals extrapolated from germination results between open and understorey microhabitats in two parapatric snapdragon plant subspecies (*A. m*. *striatum* and *A. m*. *pseudomajus*) characterized by a similar elevation range by using common garden experiments at different elevations. Signals observed under one microhabitat systematically differed in the other. Most scenarios could be inferred, with signals either shifting, appearing or disappearing between different environments. Our findings imply that caution should be taken when extrapolating the evolutionary significance of these types of experimental signals because they are not stable from one local environmental condition to the next. Forecasting the ability of plants to adapt to environmental changes based on common garden and reciprocal transplant experiments must account for the multivariate nature of the environment.

## Graphical abstract

Potential signals of adaptation to elevation obtained in snapdragon plants (*Antirrhinum majus*) common garden experiments differ between microhabitats (open habitat and understorey), thereby suggesting a lack of reproducibility of adaptation signals obtained in simplified experimental environments.

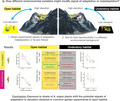

## 1 INTRODUCTION

Our ability to investigate adaptation presents a number of complications resulting from the multidimensional complexity of the environment. Awareness on this issue was recently raised in the scientific literature (Anderson & Wadgymar, 2020; Chevin & Lande, 2015; Westneat et al., 2019). From a practical perspective, the adaptation or maladaptation of populations to their habitat is generally inferred on the basis of their genetic differentiation for fitness‐related traits (Hereford, 2009; Leimu & Fischer, 2008). Traditionally, the signals indicating these mechanisms (or lack thereof) are identified through common garden experiments or reciprocal transplants, where population fitness proxies are compared between populations originating from different habitats (Kawecki & Ebert, 2004). These experiments often require to compare plant populations in a simplified environment for technical feasibility reasons. Whether this experimental simplification of population natural environments might alter our ability to infer adaptation or maladaptation remains poorly tested. In *Boechera stricta*, modifying one environmental parameter (snow cover) to simulate ancient, current and future environmental conditions driven by climate changes was enough to modify the signals indicating local adaptation and reveal a complex history of adaptive evolution (Anderson & Wadgymar, 2020). Thus, the reproducibility of signals used to infer adaptation may be challenged by the simple change of an environmental condition.

Common garden or reciprocal transplant experiments along elevation gradients have long been used to investigate climate related signals of adaptation (Halbritter et al., 2018; Lortie & Hierro, 2021). For example, if populations exposed to the climate conditions of their habitat of origin outperform populations exposed to foreign conditions, this experimental signal can be used to infer adaptation (‘local vs. foreign’ criterion defined by Kawecki & Ebert, 2004), obtained here by comparing fitness‐related traits between sites at different elevations. The results of these studies have obvious implications for our understanding of climate change consequences. From the search of 50 empirical studies published since 2017 investigating adaptation to elevation (see Table S1 for search criteria), six varied the environment (four modified biotic interactions) within the elevation. These approaches are typically carried out in open habitat conditions under full light. These environmental conditions are a valid representation of the natural environment of alpine plant species occurring exclusively above tree line (Körner, 2003). However, most plants inhabit heterogeneous habitats where several environmental conditions vary, including light, temperature, water availability, microbiota and allelochemicals. For example, approaches conducted solely in open habitats under full light neglect the possibility that shade may affect fitness‐related traits and therefore modify the experimental signal used to conclude on their adaptation, maladaptation or lack thereof. Shade is known to elicit strong phenotypic plastic changes at every life stage (including seed germination) in plants (Baskin & Baskin, 2014; Callahan & Pigliucci, 2002; Schmitt, 2003). Whether replicating an experiment at a similar elevation, even in a similar location, but in a different microhabitat (e.g. open habitat vs understorey) might affect trait values, results and conclusions on adaptive mechanisms is unknown. Thus, approaches conducted in open habitats under potentially suffer from experimental simplification. Microhabitat variability (open habitat vs understorey) is therefore ideal for investigating the reproducibility under different environments of experimental signals recorded in open habitats that are typically used to infer adaptation, maladaptation or their lack thereof.

Here, we tested for the reproducibility of results gathered from common garden experiments conducted in two different microhabitats (open habitat versus understorey). We conducted common garden experiments at high and low elevation on snapdragon plants (*Anthirrinum majus* L.) originating from different elevations. Each common garden experiment was conducted in an open microhabitat under full light and was reproduced in another type of microhabitat under the shade of the canopy of understorey trees, where the temperature is usually lower and water availability usually higher. This is a proof of concept study where the focus was made on two germination‐related traits—seed germination and timing of germination—widely recognized for their role in plant adaptation (Donohue et al., 2010). Seed germination is a direct measure of plant survival (monotonically linked to fitness). The timing of seed germination is differentially affecting fitness at low and high elevation where optimal times to germination may differ (Wagner & Simons, 2009). The timing of seed germination drives the seasonal exposure of seedlings to potentially lethal or advantageous environments for subsequent growth and reproduction (Donohue et al., 2010). At low elevation, early germination is favoured by selection for increased fecundity via a larger size before reproduction and a longer reproductive period and reduced summer drought mortality (Hoyle et al., 2015). Although germination in mountain habitats can be more complex, the high risk of seedling mortality due to adverse spring conditions at high elevation can only select for later germination than at low elevation (Giménez‐Benavides et al., 2005; Hoyle et al., 2015; Körner, 2003; Wagner & Simons, 2009). Multiple observations in southern France of snapdragon seedlings early in spring at low elevation and later in the summer at high elevation in the Pyrenees support this consensus adaptive scenario. A unique seasonal peak of germination across years is documented in snapdragon cultivated plants (Bhargava et al., 2015; Kang & Choi, 2006). Furthermore, snapdragon plants exhibit a strong phenotypic plasticity in response to shade and its suite of covarying environmental factors, for example temperature, water availability (Gourcilleau et al., 2019; Mouginot et al., 2021; Mousset et al., 2021), including in their seed germination that is delayed up to several days or prevented by shade (Kang & Choi, 2006; Smith & Whitelam, 1997). Germination sensitiveness to light is common in small‐seeded species (Baskin & Baskin, 2014). In terms of ecological significance, this might be explained by seed dispersal, with snapdragon seeds dispersed only on or near soil surface but not buried in the soil (Bhargava et al., 2015; Kang & Choi, 2006; Leishman et al., 2000; Milberg et al., 2000).

We replicated this approach in the two parapatric yet genetically closely related snapdragon subspecies (*A. m*. ssp. *striatum* and ssp. *pseudomajus*) that inhabit closely similar ecological niches in the south of France and are characterized by a similar elevation range (Khimoun et al., 2013). This is a relevant study system to address our question because the necessary prerequisites are already documented: (a) Common garden experiments conducted in an open habitat under full light revealed that elevation is suspected to be involved in the genetic divergence of phenotypic traits in *A. m*. *striatum* populations but not in the *pseudomajus* subspecies (Marin et al., 2020). (b) The large majority of populations of both snapdragon subspecies inhabit heterogeneous habitats with mixed open and understorey microhabitat conditions (Khimoun et al., 2013). Here, we focussed on 15 populations (seven *A. m*. *striatum* populations and eight *A. m*. *pseudomajus* populations) inhabiting exclusively heterogeneous habitats occurring between 6 and 1564 m (Table S2). Our hypothesis is that the type of signal typically used to infer adaptation to elevation, maladaptation or their lack thereof can change between different environmental conditions (open habitat vs understorey). We chose the ‘local elevation vs. foreign elevation’ criterion of local adaptation as signal (Kawecki & Ebert, 2004). Here, we tested for this hypothesis by comparing fitness‐related traits between two sites at different elevations.

## 2 MATERIAL AND METHODS

### 2.1 Study system

*Antirrhinum majus* L. (Plantaginaceae) is a hermaphroditic, self‐incompatible, short‐lived perennial species producing inflorescences and small seeds dispersed by gravity a few metres apart from the plant (Andalo et al., 2010; Khimoun et al., 2011). It is known to have a poor and slow rate of seed germination (Bhargava et al., 2015), with seeds germinating better on the surface of soil and at mild temperature (around 20°C, Kang & Choi, 2006).

**FIGURE 1:**
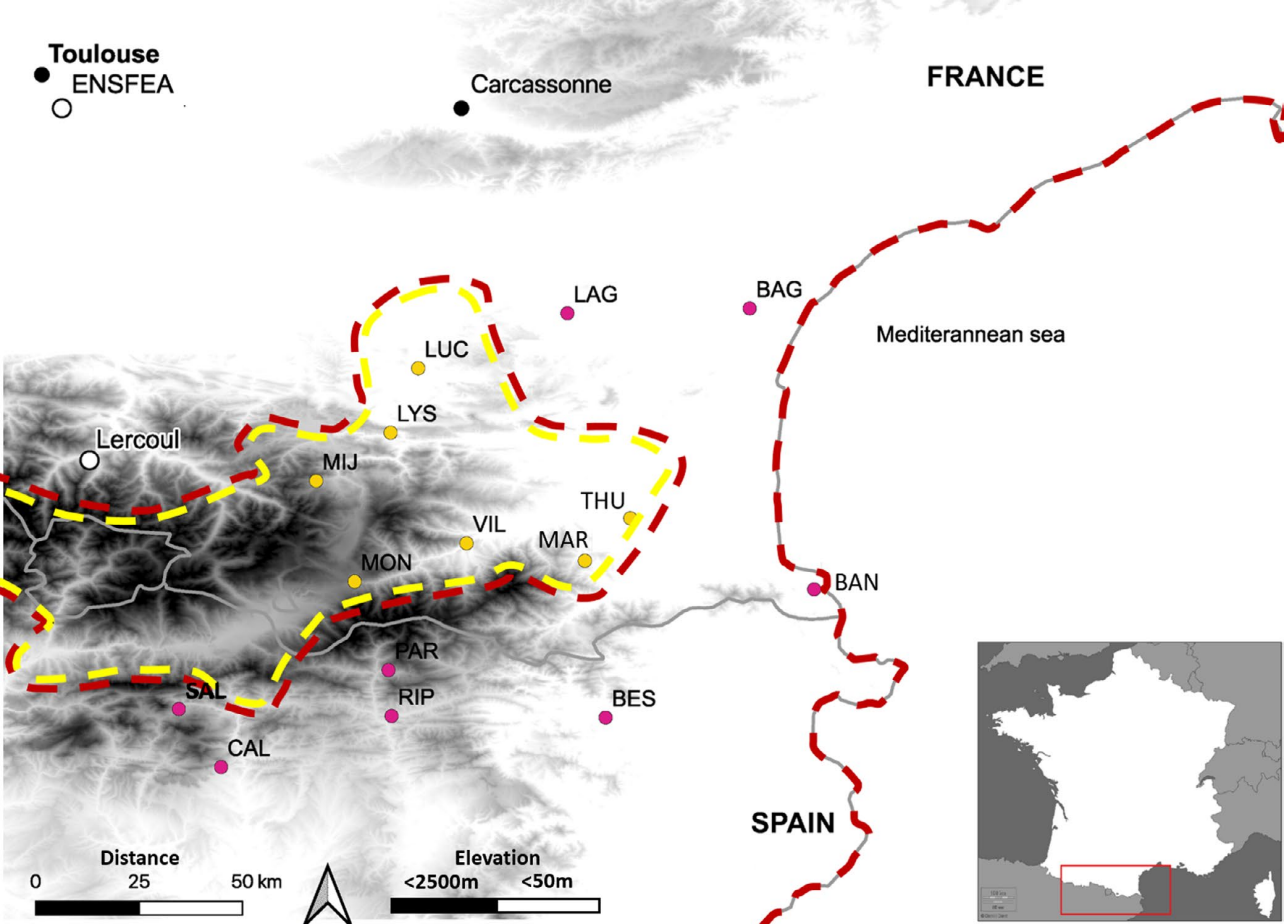
Map of *Antirrhinum majus* populations that were sampled across the geographic range of the species in Southern France. Pink dots and red dash line represent *A. m*. *pseudomajus* populations and range limit, and yellow dots and dash line represent *A*. *m. striatum* populations and range limits. Lercoul (elevation: 1100 m) and Toulouse (elevation: 152 m) are the locations of the common garden experiments (which climate conditions are described in Figure S1). Elevation follows a continuous range with lowest elevation in white (<50 m) and higher elevation in black (>2500 m)

The geographic distribution of *A*. *majus* is restricted to southern Europe (Figure 1), over the eastern half of Pyrenees Mountains, and extending south and north along the Mediterranean coast from Barcelona to Montpellier (Durán‐Castillo et al., 2021; Khimoun et al., 2013). It occurs from sea level to an altitude of 1900 m (Andalo et al., 2010), on limestone or siliceous substrates and in habitats with contrasted moisture regimes (rainfall 500–1000 mm per year). *A*. *majus* thrives in disturbed habitats and is especially common along roadside and railway embankments (Khimoun et al., 2013). *A*. *majus* plants grow in a variety of environments, including Mediterranean scrubland, scree, understorey vegetation, grassland meadows and sparse shrubland (Khimoun et al., 2013).

### 2.2 Subspecies

Snapdragon plants harbour either magenta or yellow flowers, which can be used to distinguish between the two interfertile subspecies *A. m*. *pseudomajus* and *A. m*. *striatum* respectively (Andalo et al., 2010; Durán‐Castillo et al., 2021). These subspecies are not always recognized by taxonomists (Sutton, 1988) but widely acknowledged by population and evolutionary biologists (Durán‐Castillo et al., 2021; Liberal et al., 2014; Otero et al., 2021). They are distributed parapatrically (Khimoun et al., 2011) As illustrated on Figure 1, the geographic range of *A. m*. *striatum* is surrounded by the range of *A. m*. *pseudomajus* (Khimoun et al., 2013). Contact zones can occur over a very short distance (<1 km, Whibley et al., 2006). There is evidence for gene exchange between *A. m*. *pseudomajus* and *A. m*. *striatum* (Khimoun et al., 2011), and their genetic differentiation at putatively neutral microsatellite loci is weak (ca. 1%) and one order of magnitude lower than the 10% differentiation found amongst populations (Pujol et al., 2017). Genome scans across a contact zone also revealed little differentiation between these subspecies, with the exception of loci underlying flower colour (Tavares et al., 2018; Whibley et al., 2006).

At the environmental level, the separated geographic distribution of *A. m*. *pseudomajus* and *A. m*. *striatum* is not explained by habitat differences. There is a substantial overlap of environmental conditions between the two subspecies (Khimoun et al., 2013). Both subspecies are pollinated by the same large generalist pollinators; essentially bumblebees and carpenter bees but their separation can be explained by frequency‐dependent selection operated by pollinators. Pollinators counter select the rarest type by favouring plants harbouring the same flower colour in a given area and by avoiding the white flowers of hybrid plants (Tastard et al., 2012). Phenotypic differentiation excluding flower colour is very low between these two subspecies (c.a. 2%; Marin et al., 2020).

### 2.3 Populations and seed collection

Fifteen wild populations of *A*. *majus* (seven *A. m*. *striatum* populations and eight *A. m*. *pseudomajus* populations) were sampled in 2011 (Figure 1). We used the same populations as in Marin et al. (2020), completed by one population for *A. m*. *striatum* (VIL see, Figure 1). Their locations cover most of the geographic range of the species. In each subspecies, their elevation range spans over most of the range of the species (Table S2). Climate conditions in these populations (mean annual temperature and average monthly rainfall) ranged from 14.8°C and 52 mm (at BAN, 61 m above sea level) to 6.1°C and 94 mm (at MON, 1564 m above sea level). These climate conditions segregate best in two elevation strata (6–750 and 750–1800 m) where climate conditions differ between strata but are consistent amongst sites within strata (Marin et al., 2020) based on fifty‐year averages (1950–2000) extracted from the WorldClim database (resolution 1 km², www.worldclim.org, Hijmans et al., 2005). Data and methods used by Marin et al. (2020) to establish these strata are freely accessible on the ZENODO [Open Aire EU official] repository: https://zenodo.org/record/5503746.

Seed families used in this experiment were not sampled directly in the wild but produced by two successive generations of parental plants that were germinated in controlled conditions and grown in a common garden environment (see Figure S2 and associated text for the detailed cultivation protocol and conditions). Only the first parental generation of plants was germinated from seeds collected from field populations on mature plants. These two generations of plants regenerated before our experiment reduced potential maternal environmental effects differentially affecting populations that could have otherwise biased the trait values recorded during the experiments presented here. The growing of plants for two generations in a common garden might nevertheless has resulted in the preconditioning of plants to this specific set of environmental conditions, which might homogenize phenotypic difference between populations. This potential homogenization effect would only partly be an issue because it could only make the finding of trait genetic differentiation more conservative.

### 2.4 Experimental design in the common gardens

We sowed 5360 seeds in spring 2015 on the first days of June. Seeds germinated and seedlings were grown outdoor in the common gardens surrounded by fences. In every garden, the 15 study populations (and the 15 sibship families within each population) were the same, so that the four gardens (open habitat and understorey in both low‐ and high‐elevation sites) were composed by a similar gene pool. In every garden, every seed family was represented by six or seven seeds (Table S2). This experimental setting allows to compare the effect of elevation with a focus on climate conditions while homogenizing many other potential effects (e.g. soil composition). Seeds were sown on the top of individual pots (9 × 9 × 10 cm) with clay universal (TS3 Argile code 404, Klasmann©) and compost universal (BP2 Kompact code 294, Klasmann©). These pots were arranged in a randomized design over a few square metres on a tarpaulin covered with compost universal. Plants were grown in pots filled with no nutrient addition and under outdoor climatic conditions in planting sites. Plants were supplied with water in case of prolonged drought.

### 2.5 Common garden sites representing low‐ and high‐elevation climate conditions

Seeds from every population originating from each of the low‐ (from sea level to 750 m) and high‐elevation strata (from 750 to 1600 m) were set to germinate in two sites (Figure S2). One site was located at low elevation at ENSFEA school campus, Toulouse, France (elevation 152 m). The other site was located at high elevation, in the Siguer valley at Lercoul, France (elevation 1100 m; see Figure 1). These two sites were chosen because their climatic conditions were respectively representative of the average climatic conditions typically experienced by populations in the low (from sea level to 750 m) and high (from 750 to 1800 m) elevation strata. For instance, the site at high elevation received more rainfall, was cooler and had a less severe summer drought than the low‐elevation site (See supplementary information, Figure S1). Keeping in mind that sites were chosen for their correspondence with the average climate conditions of the two elevation strata, one limitation of our experiment is that the effects of site and elevation were confounded. Although we homogenized many environmental conditions (e.g. germination soil substrate, root development available space) between sites as to focus on climate differences between high and low elevation, differences between elevations cannot be attributed exclusively to elevation and could also be due to other site‐specific uncontrolled environmental conditions. This lack of site replication at a given elevation is not really an issue in this study because it has no effect on our ability to test for the reproducibility of the signal between different microhabitats.

### 2.6 Microhabitat variation: open habitat versus understorey

Our aim was not to evaluate the role of each microhabitat in snapdragon adaptation, which would have required a far more complex experimental set up, but to test for the reproducibility of adaptive signals between different microhabitats (open habitat vs. understorey). On each site, seeds were exposed to the two different microhabitats: open habitat under full light and understorey trees providing shade. We used natural vegetation to induce differential environmental conditions based on the presence and absence of understorey trees: common spruce (*Picea abies*), silver fir spruce (*Abies alba*), beech (*Fagus sylvatica*) etc. at high elevation and European chestnut (*Castanea sativa*), common hazel (*Coryllus avellana*), European filbert (*Cornus sanguinea*) etc. at low elevation. On each site, the common gardens in different microhabitats (open habitat and understory) were within 200 m of each other to keep the local environmental conditions that are not affected by microhabitat variation as similar as possible. Along with the shade induced by understorey vegetation, soil moisture, temperature and biotic interactions (e.g. microbiota and allelochemicals) were undoubtedly different between the two types of microhabitat. Open and understorey microhabitats were artificially separated in our experimental design, whereas in nature, snapdragon plants germinate and develop in heterogeneous habitats where both these types of conditions are mixed. Differential adaptation to these microhabitats between populations was therefore not expected. We estimated the contrast between microhabitat types by measuring the photosynthetically active radiation (PAR) in each common garden, which was strongly and significantly reduced in the understorey (Figures S3 and S4). Small changes in PAR are expected with elevation alongside with temperature, rainfall etc. Although some differences in PAR occurred between high and low elevations in our experiment, they were negligible in comparison to the shading effect of understorey trees which was stronger by several orders of magnitude (Figure S3).

### 2.7 Germination‐related traits

Here, we focussed on two germination‐related traits: seed germination (whether a seed germinated or not) and the time to germination (number of days between sowing and germination, the latter corresponding to the first time any part of a seedling was spotted above ground). We monitored germination in common gardens three times per week during the spring and summer 2015 (from June to October). In accordance with the current knowledge on the species, we considered that seeds not germinating during the first year harboured no potential for germination in future years and did not play a role in a particular ecological strategy of delayed germination across seasons.

### 2.8 Statistical analyses

We performed GLMMs (generalized linear mixed models) on germination‐related traits for each subspecies separately. The fixed effects included the site elevation, the elevation of origin of the populations (as a discrete variable; ‘high’ vs. ‘low’), the microhabitat ‘treatment’ (‘open habitat’ vs. ‘understorey’) and their interactions. The population and the family were included as random effects. Traits were modelled differently depending on their distribution: (a) germination (for each individual 0 vs. 1) was modelled as a binomial variable (with a logit link function) and (b) the time to germination was modelled as a Gaussian variable (normality assumptions were verified). We presented 95% confidence intervals and used their lack of overlap with zero or lack of overlap between treatments to define what effects were statistically significant in our GLMM approaches, and this is a conservative approach that provides information on the statistical effects (range, direction, strength and reliability) that is not provided by *p* values (Ho et al., 2019).

The global GLMM approach tells us all we need to know about a multifactorial analysis of adaptation signals in a ‘local vs. foreign’ approach. The crossing of reaction norms (*i*.*e*. phenotypic responses of same genotypes between high‐ and low‐elevation gardens) of populations originating from high‐ and low‐elevation habitats is the adaptive signal for measurements monotonically related to fitness. Since our aim was not to assess adaptation but to evaluate the potential modification of this type of adaptive signals by an additional environmental variable, we also tested for interaction effects with the microhabitat. We also represented and tested for the significance of effects analysed in isolation; for example high vs low elevation of origin within each garden by using Wilcoxon tests (non‐parametric data). We checked that the results were consistent across populations originating from a same elevation (Figures S5 and S6). Finally, we compared whether the ‘local vs foreign’ results were implying a similar signal of adaptation, maladaptation or their lack thereof in different microhabitats in order to evaluate the reproducibility.

All statistical analyses were performed using the R.3.5.0 software (R Core Team, 2018). All generalized mixed models were implemented in R via the glmmTMB R‐package (Brooks et al., 2017). Assumptions of models (e.g. heteroscedasticity, over‐ or under‐dispersion, significance of outliers) were checked both visually and by using the DHARMa R‐package (Hartig, 2020). The models for the time to germination in *A. m*. *pseudomajus* violated uniformity of residuals assumptions according to DHARMa analysis. Although the visual check of the model assumptions would have considered the assumptions were respected, caution must be taken when considering the results of this model.

## 3 RESULTS

### 3.1 Statistical significance of ‘local vs foreign’ effects and microhabitat effects

In both subspecies, elevation explained a significant part of variance for both germination‐related traits (Table 1). The significant effects of three‐way interactions including the elevation of origin, the site elevation and the microhabitat show that the signal of either adaptation, maladaptation or their lack thereof differed between microhabitats (Table 1).

**TABLE 1:** Results from the generalized linear mixed models (GLMMs) testing the effects of site elevation, elevation of origin, microhabitats and their interactions on germination‐related traits in both subspecies of snapdragon plants

	*A. m*. *striatum*	*A. m*. *pseudomajus*
Germination (binomial)	Marginal *R* ^2^ = 0.12, conditional *R* ^2^ = 0.17	Marginal *R* ^2^ = 0.13, conditional *R* ^2^ = 0.22
Fixed effects	Estimate (CI 95%)	Estimate (CI 95%)
Intercept	**−0.54 (−0.87, −0.21)**	**−0.93 (−1.23, −0.63)**
Site elevation (low)	**−1.63 (−2.03, −1.22)**	**−1.75 (−2.21, −1.29)**
Elevation of origin (low)	−0.46 (−0.97, 0.05)	−0.01 (−0.44, 0.42)
Microhabitat (understorey)	**−0.71 (−1.05, −0.37)**	−0.15 (−0.48, 0.19)
Origin*site	0.07 (−0.59, 0.74)	0.20 (−0.44, 0.84)
Site*understorey	**1.32 (0.77, 1.87)**	**1.53 (0.95, 2.11)**
Origin*understorey	**1.27 (0.77, 1.77)**	**0.67 (0.21, 1.14)**
Origin*site*understorey	**−1.09 (−1.95, −0.22)**	**−1.27 (−2.08, −0.46)**
Time to germination (Gaussian)	Marginal *R* ^2^ = 0.21, conditional *R* ^2^ = 0.30	Marginal *R* ^2^ = 0.22, conditional *R* ^2^ = 0.33
Fixed effects	Estimate (CI 95%)	Estimate (CI 95%)
Intercept	**34.13 (31.52, 36.74)**	**27.82 (25.66, 29.98)**
Site elevation (low)	**−16.13 (−19.9, −12.36)**	**−13.09 (−17.29, −8.89)**
Elevation of origin (low)	**−5 (−9.15, −0.85)**	**4.97 (1.92, 8.01)**
Microhabitat (understorey)	**−8.41 (−11.39, −5.43)**	**−2.95 (−5.7, −0.21)**
Origin*site	0.65 (−5.8, 7.1)	−5.36 (−11.19, 0.47)
Site *understorey	**10.96 (5.7, 16.21)**	**6.37 (1.25, 11.49)**
Origin*understorey	4.34 (−0.01, 8.68)	**−6 (−9.71, −2.3)**
Origin*site*understorey	1.77 (−6.6, 10.14)	**7.31 (0.17, 14.52)**

Note

The modality of the explanatory variable that is associated with the parameter estimate is indicated between brackets. Population and the family were taken into account by being included as random effects. Marginal *R* ^2^ is the variance explained by fixed effects. Conditional *R* ^2^ is the part of variance explained by both fixed and random effects. Parameter estimates are given with their 95% confidence interval between brackets. Significant effects (no overlap between 95%CI and zero) are indicated in bold. Interaction terms are contracted as follows: origin: elevation of origin (low), site: site elevation (low), understorey: understorey microhabitat (understorey).

### 3.2 Germination potential adaptive signal in the open microhabitat

**FIGURE 2:**
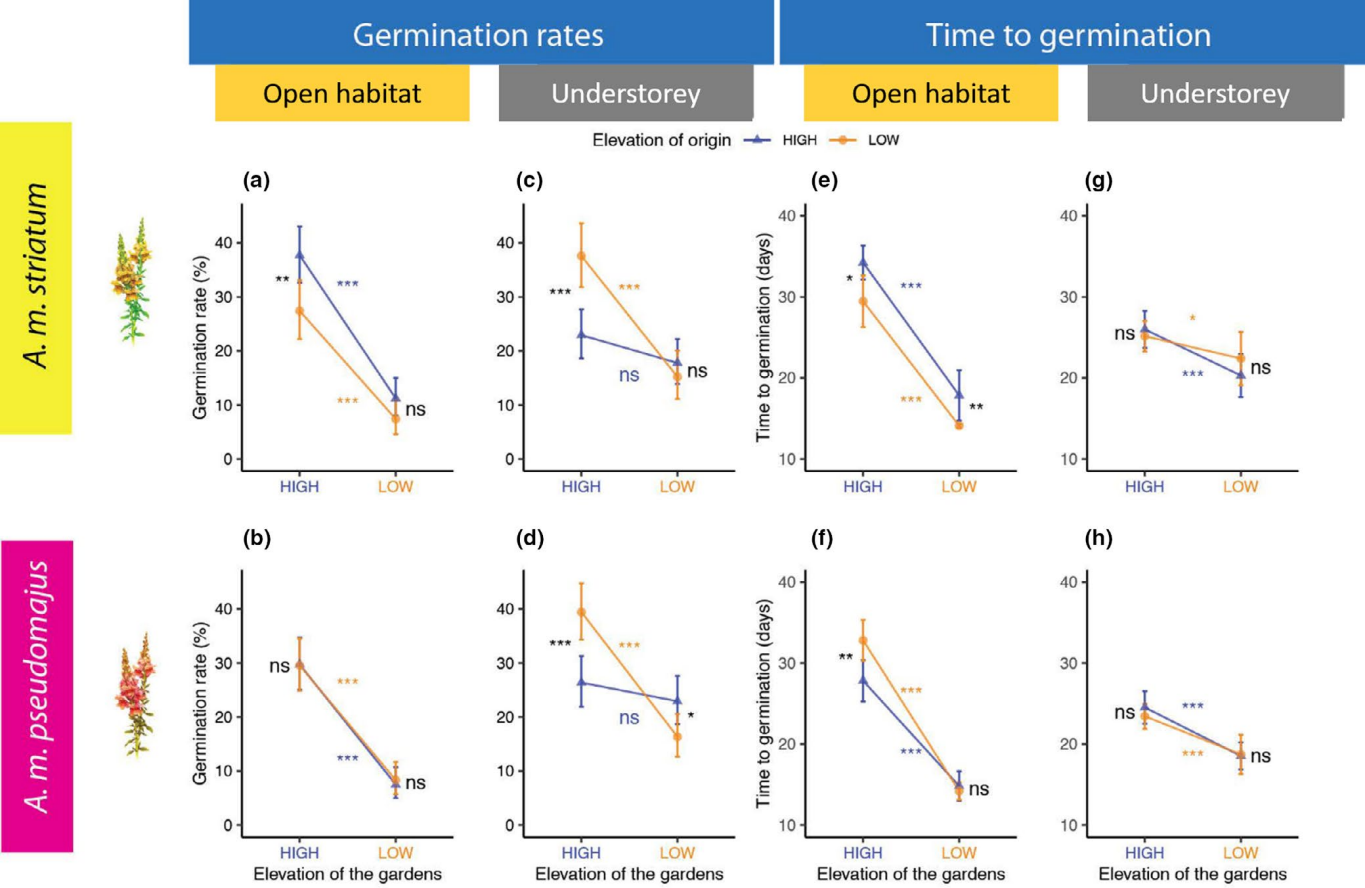
Reaction norms of germination‐related traits (mean values ±95% CI) for seven populations of *A. m*. *striatum* and eight populations of *A. m*. *pseudomajus* in the two sites (low and high elevation) and in the two microhabitats per site (open habitat and understorey). The upper panel graphs represent *A. m*. *striatum* reaction norms. The lower panel graphs represent *A. m*. *pseudomajus* reaction norms. Line colours indicate elevations of origin (orange for low and blue for high), and colours on the x‐axis represent the elevation of the gardens (orange for low and blue for high). Significant differences are indicated by asterisks. ***: *p* value ≤ 0.001, **: 0.001 < *p* value ≤ 0.01, *: 0.01 < *p* value ≤ 0.05. Lack of significance is indicated by ‘ns’: *p* value ≥ 0.5 Asterisks or ns symbols at the ends represent the difference or lack of difference between populations originating from different elevations in a similar garden and therefore exposed to a similar elevation. Asterisks or ns symbols on the lines represent the difference or lack of difference between common gardens at different elevations for populations originating from a similar elevation

In the subspecies *A. m*. *striatum*, the ‘local vs. foreign’ criterion of adaptation to the climate conditions corresponding to the elevation of origin of the populations was partially satisfied for seed germination in the open microhabitat under full light. This type of adaptation signal was partly identified in the high‐elevation site but not in the low‐elevation site. In the high‐elevation site, seeds originating from high‐elevation habitats germinated more than seeds originating from low‐elevation habitats (Figure 2a). Yet, in the low‐elevation site, differences in germination rate were not significant between populations from high and low‐elevation habitats (Figure 2a). In the subspecies *A. m*. *pseudomajus*, in the open microhabitat, no evidence for genetic differentiation was found in terms the germination rate (Figure 2b).

### 3.3 Germination signal in the understory microhabitat under the shade of trees

We obtained contrasted results at high‐elevation site for *A. m*. *striatum* in the understorey microhabitat under the shade of trees; seeds from high‐elevation habitats germinated less (Figure 2c). In the low‐elevation site, results were no different in the understorey microhabitat; seeds from high‐ and low‐elevation habitats had similar germination rates. In the subspecies *A. m*. *pseudomajus*, we obtained contrasted results between the open microhabitat where no effect of the elevation of origin was found and the understorey. In the understorey microhabitat, the local vs foreign criterion suggested a maladaptation signal (Figure 2d). This is because seeds from low‐elevation habitats germinated more in the high‐elevation site, and in the low‐elevation site, seeds from high‐elevation habitats germinated more.

### 3.4 Time to germination signal in the open microhabitat

In the subspecies *A. m*. *striatum*, a signal of genetic divergence on the time to germination was identified between the high‐ and low‐elevation sites in the open microhabitat under full light. In the high‐elevation site, seeds from high‐elevation habitats germinated later than seeds from low‐elevation habitats (Figure 2e). Seeds originating from low‐elevation habitats germinated significantly earlier than seeds originating from high‐elevation habitats in the low‐elevation site (Figure 2e). This type of partial signal is coherent between open microhabitat sites because germinating earlier at lower elevation is widely acknowledged to be advantageous (Hoyle et al., 2015; Leger et al., 2009). In the subspecies *A. m*. *pseudomajus*, a signal of genetic differentiation was observed exclusively in the high‐elevation site but not in the low‐elevation site. In the high‐elevation site, seeds from high‐elevation habitats germinated earlier than seeds from low‐elevation habitats (Figure 2f). As for *A. m*. *striatum*, caution must be taken when interpreting this partial signal as potentially adaptive or maladaptive.

### 3.5 Time to germination signal in the understorey microhabitat

In both subspecies, we obtained contrasted results between microhabitats. In the understorey microhabitat under the shade of trees, we found no signal of genetic differentiation on the time to germination between populations originating from high‐ and low‐elevation habitats (Figure 2g and h).

### 3.6 Changes between microhabitats in the ‘local vs foreign’ signal

**FIGURE 3:**
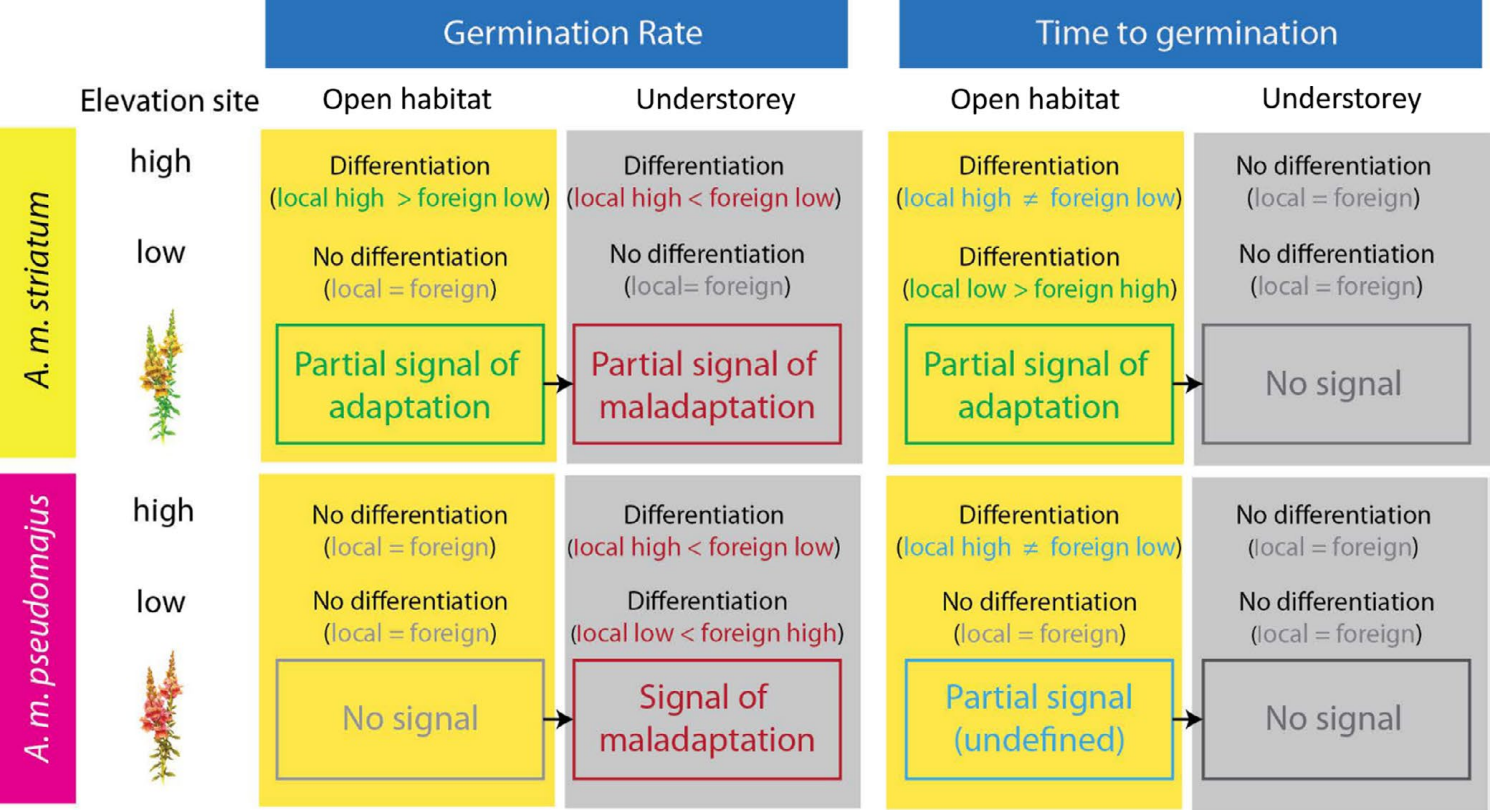
Summary diagram illustrating changes in signals of adaptation or maladaptation to elevation between microhabitats (open microhabitat and understorey), for two germination‐related traits, in two subspecies of *Antirrhinum majus*

All the contrasted signals observed between microhabitats are synthesized on Figure 3. Modifications were found in nearly all directions with a coherent negative effect of the understorey microhabitat on germination‐related traits where no potentially adaptive signal was obtained.

## 4 DISCUSSION

### 4.1 Non‐reproducibility of signals between microhabitats

Our results showed that the type of signals used to infer adaptation to elevation, maladaptation or their lack thereof, which are systematically assessed in open habitats under full light in plant common garden and reciprocal transplant experiments, changed under the shade of understorey trees in snapdragon plants (Figure 3). We observed nearly every possible scenario. For example, the signal in *A. m*. *striatum* for the germination rate reversed from a partial signal of adaptation to maladaptation. We also observed the signal disappearing in the understorey microhabitat in both subspecies for the time to germination. Finally, we even observed that a maladaptive signal appeared in the understorey microhabitat in *A. m*. *pseudomajus* for the germination rate. Our results showed that a simple environmental change of microhabitat in the same location can generate critical shifts in the signal that can substantially modify conclusions on adaptive mechanisms. This lack of reproducibility in a different environmental background implies that the experimental simplification of environments that are complex in nature can bias our ability to infer adaptation or maladaptation.

### 4.2 The multidimensional complexity of the environment

Although the shade of understorey trees likely induced less germination and delayed germination as expected (Smith & Whitelam, 1997), results in the understorey microhabitat could not be inferred from simply scaling down the results found in the open microhabitat under full light in our study. One advantage of our protocol was to use seed from similar sibship families to go beyond exposing plants from the same populations to different conditions and replicate the exact same gene pool in different microhabitats characterized by different environmental conditions (e.g. shade, temperature, water availability, microbiota and allelochemicals). The shift in signals that we observed was therefore the result of phenotypic plasticity and could not be biased by population genetic heterogeneity. Our findings corroborate the recent awareness in the theoretical scientific literature of the non‐intuitive impact of environmental complexity on phenotypic plasticity and adaptive scenarios (Chevin & Lande, 2015; Westneat et al., 2019). We observed a disparity in the plastic response of a single trait to elevation and microhabitat variation, which is likely due to the multiple processes underlying plasticity in germination in response to change in multiple environment factors underlying elevation and microhabitat effects on the phenotype (Morel‐Journel et al., 2020). Our results suggest potential interactions between the plastic responses to changes in these environments that can render the adaptive responses of plants living in complex environments hardly predictable in a simplified environment where microhabitat variation is not taken into account. Empirical studies testing for the stability of signals indicating plant adaptive responses across complex environments are rare. More studies are needed such as this one and Anderson and Wadgymar (2020) to assess how signals of adaptation to elevation are disturbed by changes in environmental variables leading to observe signs of maladaptation. Here, we call for more studies on this topic to assess whether neglecting the multivariate nature of the environment might lead to incorrect assessments of species adaptive history and potential.

### 4.3 Implications for snapdragon plants

Ignoring the results obtained in the understorey microhabitat could lead to mistakenly interpret partial signals obtained in the open microhabitat under full light as being consistent with a scenario of adaptation of *A. m*. *striatum* plants to elevation, incidentally corroborating former results from a *Q*_ST_‐*F*_ST_ comparison of experimental populations also conducted in an open habitat (Marin et al., 2020). In our study, a signal was deemed ‘partial’ when trait genetic differentiation was observed in one garden, but not the others. For example, the germination rate of *A. m*. *striatum* populations originating from high‐elevation habitats was only higher in the high‐elevation site in the open microhabitat under full light (Figure 3). At low elevation, we found a lack of difference six times out of eight (Figure 3). Experimental artefacts can mask a signal of adaptation or maladaptation in some sites (Kawecki & Ebert, 2004). One cannot exclude the possibility that hot and dry climatic conditions in low‐elevation gardens homogenized seed responses. We also defined a signal as ‘partial’ for the time to germination when genetic differentiation was observed in the two sites because the germination time providing the best fitness could not be clearly identified at high elevation (Figure 3). Knowledge on the fitness landscape for the time to germination at high elevation would be required to obtain a complete signal. Partial signals may suggest an imprint of natural selection because *A. m*. *striatum* populations originating from high‐elevation habitats outperformed the populations originating from low‐elevation habitats in the high‐elevation site via higher germination rates and the populations originating from low‐elevation habitats outperformed the populations originating from high‐elevation habitats in the low‐elevation site via delayed germination. This incomplete pattern is not convincing evidence for adaptation to elevation derived from the ‘local vs foreign’ criterion (as defined by Kawecki & Ebert, 2004). Our main finding is that results obtained in a different microhabitat under the shade of understorey trees totally contradict this incomplete adaptive scenario. In the absence of this finding, results found in the open microhabitat under full light would have invited follow‐up studies to detect a complete adaptive signal in replicated open habitat sites. Based solely on results obtained in the understorey, we would have concluded to the potential maladaptation of both subspecies to elevation. Instead, our findings have implications for snapdragon plants because potential adaptive scenarios must be regarded with extreme caution. In nature, seed of most snapdragon plant populations and all the seeds of the populations used in this study germinate under mixed open and understorey microhabitat heterogeneous environments. Our ability to extrapolate adaptive mechanisms in *A*. *majus* presents a number of complications resulting from local environmental heterogeneity. These contrasted results in different microhabitats suggest varying selection on germination traits inside populations where the vegetation cover is heterogeneous. A recent paper on adult snapdragon plant traits corroborates that fluctuating selection driven by the local environmental heterogeneity can occur at a small spatial scale within populations (Marrot et al., 2021). The simplified environments that we used in snapdragon plant experiments have improved our understanding of microhabitat variation on germination, but do not suffice to grasp the complexity of the adaptive mechanisms at play in wild populations where the variation of fitness‐related traits is shaped by multidimensional interactions between environmental factors.

## 5 CONCLUSION

Microhabitats affected the adaptive signal. Our experiment revealed the non‐reproducibility in different microhabitats (open habitat vs. understorey) of potential adaptive scenarios drawn from common garden experiments comparing multiple populations at different elevations. Opposite signals were found that suggest heterogeneous selection inside wild snapdragon populations where plants are generally exposed to mixed microhabitat conditions. Experimental signals of plant adaptation detected in common garden studies are usually obtained in open habitat under full‐light conditions. Lack of reproducibility between microhabitats is likely to affect conclusions from similar assessments conducted in other species naturally occurring in heterogeneous environments. Our findings imply that adaptive scenarios built upon the experimental simplification of the natural environment must be discussed with a lot of caution. They also imply that forecasting the ability of plants to adapt to environmental changes based on common garden and reciprocal transplant experiments must account for the multivariate nature of the environment.

## CONFLICT OF INTEREST

The authors of this article declare that they have no financial conflict of interest with the content of this article.

## AUTHOR CONTRIBUTIONS

BP designed the research programme. SM, JA, MI, GO, AlG and BP carried out the experiments; AG and PM analysed the data; AG and BP wrote the manuscript.

### PEER REVIEW

The peer review history for this article is available at https://publons.com/publon/10.1111/jeb.13973.

## Supporting information

Supplementary Material

Supplementary Material

## Data Availability

Code and data for producing figures and results in this paper are openly available on ZENODO [Open Aire EU official] repository: https://zenodo.org/record/5503746.
