# Supplementary material for: Non‐reproducible signals of adaptation to elevation between open and understorey microhabitats in snapdragon plants
Source: J Evol Biol. 2021 Dec 20;35(2):322–32. doi: 10.1111/jeb.13973 (PMC9299861; doi:10.1111/jeb.13973)
Supplement: Supplementary file 1 — Supplementary Material [file JEB-35-322-s001.docx]

supporting information for:

**Non-reproducible signals of adaptation to elevation between light and shade environments in Snapdragon plants**

Authors to be included after double blind review process

Includes:

# **supplementary material**

***Supplementary Information***

***Supplementary Table***

Table S1: First fifty studies (DOIs) investigating adaptation to elevation listed by google scholar.

Table S2: Description of *Anthirinum majus* populations grown in the two common garden experiments.

***Supplementary Figures***

Figure S1: Environmental conditions recorded monthly over a year

Figure S2: diagram representing the experimental design

Figure S3: Difference in photosynthetically active radiation (PAR) between high and low elevation sites in a) full light, and b) under the shade of understorey vegetation.

Figure S4: Differences in spectral data between high and low-elevation sites in a) full light, and b) under the shade of understorey vegetation.

Figure S5: Reaction norms of germination-related traits for seven populations of *Anthirrinum majus striatum* in the two sites (low and high elevations) and under two treatments (full light habitat and understorey shade).

*±*

Figure S6: Reaction norms of germination-related traits for eight populations of *Anthirrinum majus pseudomajus* in the two sites (low and high elevations) and under two treatments (full light habitat and understorey shade).

# **supplementary material**

- 1. ***Supplementary Information***

Environmental conditions in planting sites, i.e., Temperature (°C), Relative Humidity (%) and Light intensity (lux), were measured during the experiment at each garden site and environment using external data loggers (HOBO U12-012) with one measure per hour. Photosynthetically Active Radiation (PAR) and spectral data were measured at each planting site and environment in cloud-free conditions, to minimize the external effects of the atmospheric conditions, using a fiber-optic spectrometer (AvaSpec-2048; Avantes, Netherlands). The sensor’s field of view was 25°, with a full sky irradiance remote cosine corrector. Spectral data was measured at wavelengths ranging from 400 nm to 1000 nm at 0.6 nm intervals and flux densities of PAR were measured at 60 sec intervals every 15 days from 10 am to 2 pm during the experiment. Average measurements were calculated for each type of light measurement, each site and environment. The red:far-red (R/FR) ratio was calculated from the spectral data (photon flux between 655 and 665 nm divided by photon flux between 725 and 735 nm).

Meteorological variables and light availability were compared between planting sites, in different environments by using linear mixed-effects models. Daily average Temperature, Relative Humidity and Light intensity were compared between planting sites (included as a fixed effect) and days (included as a random effect). Differences between sites in terms of PAR and spectral data averages were determined between planting sites (included as a fixed effect) and hours or wavelengths (included as a random effect).

- 1. ***Supplementary Tables***

Table S1: First fifty studies (DOIs) investigating adaptation to elevation listed by google scholar.

Studies published since 2017. Google scholar search based on the keywords: elevation, altitude, common garden experiment, reciprocal transplant.

| Additional abiotic environment variable | https://doi.org/10.1007/s11258-021-01178-6  https://doi.org/10.1111/ele.13427 |
| --- | --- |
| Additional biotic environment variable | https://doi.org/10.1002/ece3.6709  https://doi.org/10.1111/ele.13190  http://dx.doi.org/10.1111/1365-2745.13440  https://doi.org/10.1186/s12898-018-0197-5 |
| No additional environment variable | https://doi.org/10.1080/13416979.2021.1964151  https://doi.org/10.15177/seefor.20-07  https://doi.org/10.1002/ajb2.1108  https://doi.org/10.1111/een.13087  https://doi.org/10.1186/s12898-018-0194-8  https://doi.org/10.1080/23766808.2021.1940049  https://doi.org/10.1111/evo.14109  https://doi.org/10.1007/s13595-017-0673-7  https://doi.org/10.1016/j.envexpbot.2019.103894  https://doi.org/10.1093/biolinnean/blx068  https://doi.org/10.1111/mec.15546  https://doi.org/10.1002/ece3.4999  https://doi.org/10.1371/journal.pone.0187708  https://doi.org/10.1111/1365-2435.13830  https://doi.org/10.3390/f12010069  https://doi.org/10.1038/s41467-020-15208-w  https://doi.org/10.3732/ajb.1600414  https://doi.org/10.1111/1365-2745.12955  https://doi.org/10.1093/aobpla/plx027  https://doi.org/10.1007/s10722-018-0693-7  https://doi.org/10.1186/s12863-017-0529-z  https://doi.org/10.1016/j.foreco.2017.12.014  https://doi.org/10.1080/23818107.2021.1980434  https://doi.org/10.1111/1365-2745.13171  https://doi.org/10.14214/sf.10381  https://doi.org/10.1111/evo.13521  https://doi.org/10.1016/j.baae.2017.05.003  https://doi.org/10.1111/nph.16009  https://doi.org/10.1002/ece3.6651  https://doi.org/10.1002/1438-390X.12099  https://doi.org/10.1007/s10886-019-01084-2  https://doi.org/10.2179/0008-7475.84.2.128  https://doi.org/10.1111/nph.17678  https://doi.org/10.1371/journal.pgen.1008512  https://doi.org/10.1038/s41437-020-00355-z  https://onlinelibrary.wiley.com/doi/full/10.1002/ece3.3947  https://doi.org/10.1111/ele.13169  https://doi.org/10.1111/eva.12954  https://doi.org/10.1086/702312  https://doi.org/10.3390/f10040293  https://doi.org/10.1007/s00035-018-0211-8  https://doi.org/10.1111/1365-2435.13071  https://doi.org/10.1098/rspb.2021.0077  https://onlinelibrary.wiley.com/doi/abs/10.1111/evo.13288 |

Table S2: Description of *Anthirinum majus* populations grown in the two common garden experiments.

Lat= latitude, Long= Longitude, Elev.= Elevation, Subsp.= subspecies, PS= *A. m. pseudomajus*, ST= *A. m. striatum*, Nfam= number of families, N= number of plants,

| Acronym | Location | Lat | Long | Elev. (m) | Elev. | Subsp. | Description | Nfam | Nseed |
| --- | --- | --- | --- | --- | --- | --- | --- | --- | --- |
| BAG | Bages | 43.10 | 2.98 | 6 | Low | PS | rocky shrubland | 15 | 354 |
| BAN | Banyuls-sur-Mer | 42.49 | 3.12 | 61 | Low | PS | rocky shrubland | 15 | 360 |
| BES | Besalú | 42.21 | 2.67 | 195 | Low | PS | stone walls | 15 | 363 |
| CAL | Berga | 42.10 | 1.83 | 838 | High | PS | herbaceous roadside | 15 | 361 |
| LAG | Lagrasse | 43.09 | 2.58 | 149 | Low | PS | rocky shrubland | 15 | 361 |
| LUC | Luc-sur-Aude | 42.97 | 2.26 | 227 | Low | ST | rocky shrubland | 15 | 361 |
| LYS | Pierre-Lys | 42.83 | 2.20 | 713 | Low | ST | rocky river bank | 15 | 361 |
| MAR | Saint-Marsal | 42.55 | 2.62 | 628 | Low | ST | herbaceous roadside | 15 | 360 |
| MIJ | Mijanès | 42.73 | 2.04 | 1347 | High | ST | herbaceous meadows | 13 | 312 |
| MON | Mont-Louis | 42.51 | 2.12 | 1564 | High | ST | stone walls | 15 | 363 |
| PAR | Pardines | 42.31 | 2.20 | 1118 | High | PS | herbaceous roadside | 15 | 360 |
| RIP | Ripoll | 42.21 | 2.20 | 709 | Low | PS | herbaceous roadside | 15 | 360 |
| SAL | Saldes | 42.23 | 1.74 | 1126 | High | PS | herbaceous meadows | 15 | 361 |
| THU | Thuir | 42.64 | 2.72 | 130 | Low | ST | herbaceous roadside | 15 | 362 |
| VIL | Villefranche-de-Conflent | 42.59 | 2.48 | 568 | Low | ST | rocky shrubland | 15 | 361 |

- 1. ***Supplementary Figures***


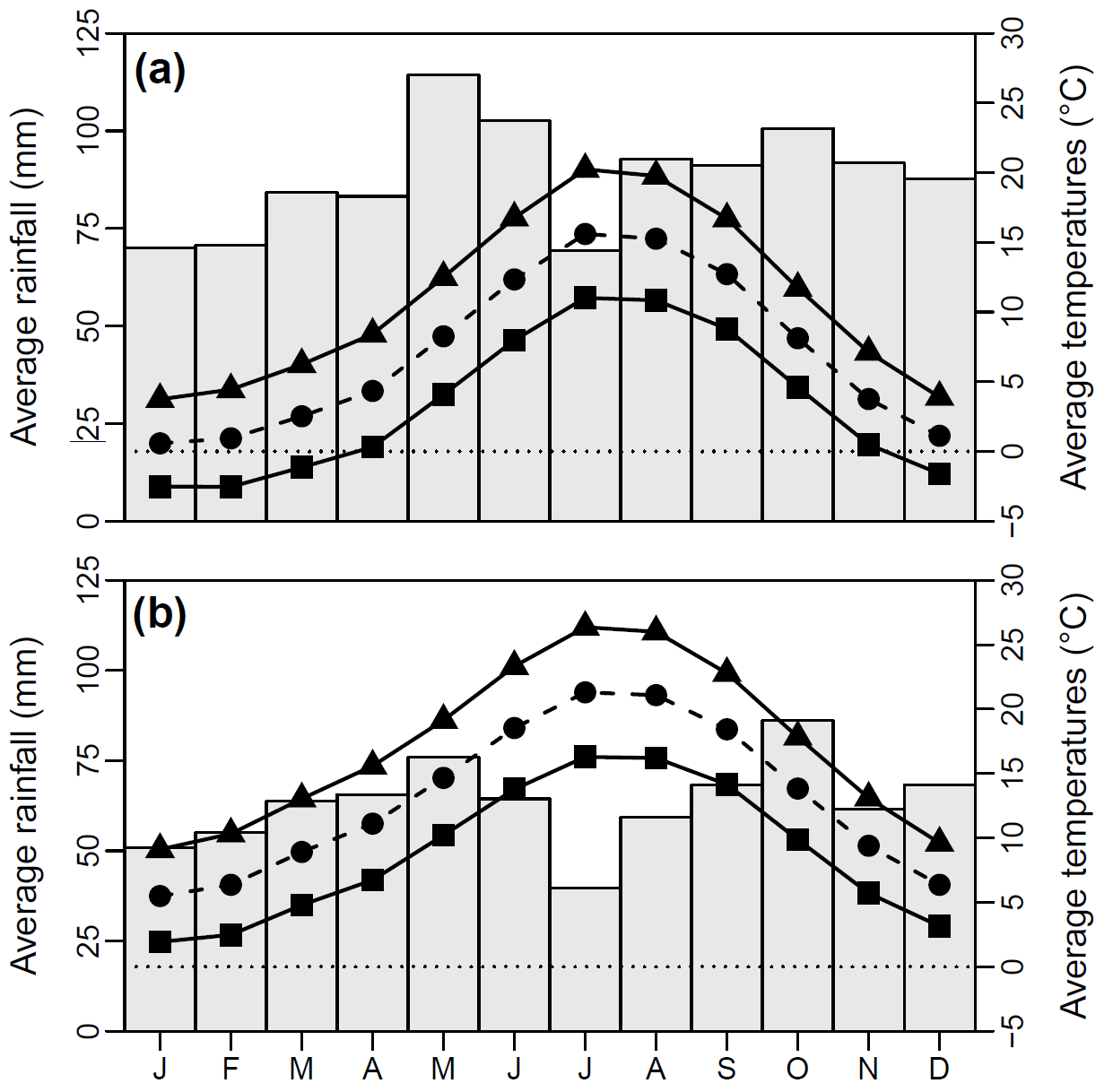


Figure S1: Environmental conditions recorded monthly over a year.

a) high-elevation and low-elevation sites. Histograms represent average rainfall, and lines average (dot points), minimum (square) and maximum (triangle) temperatures


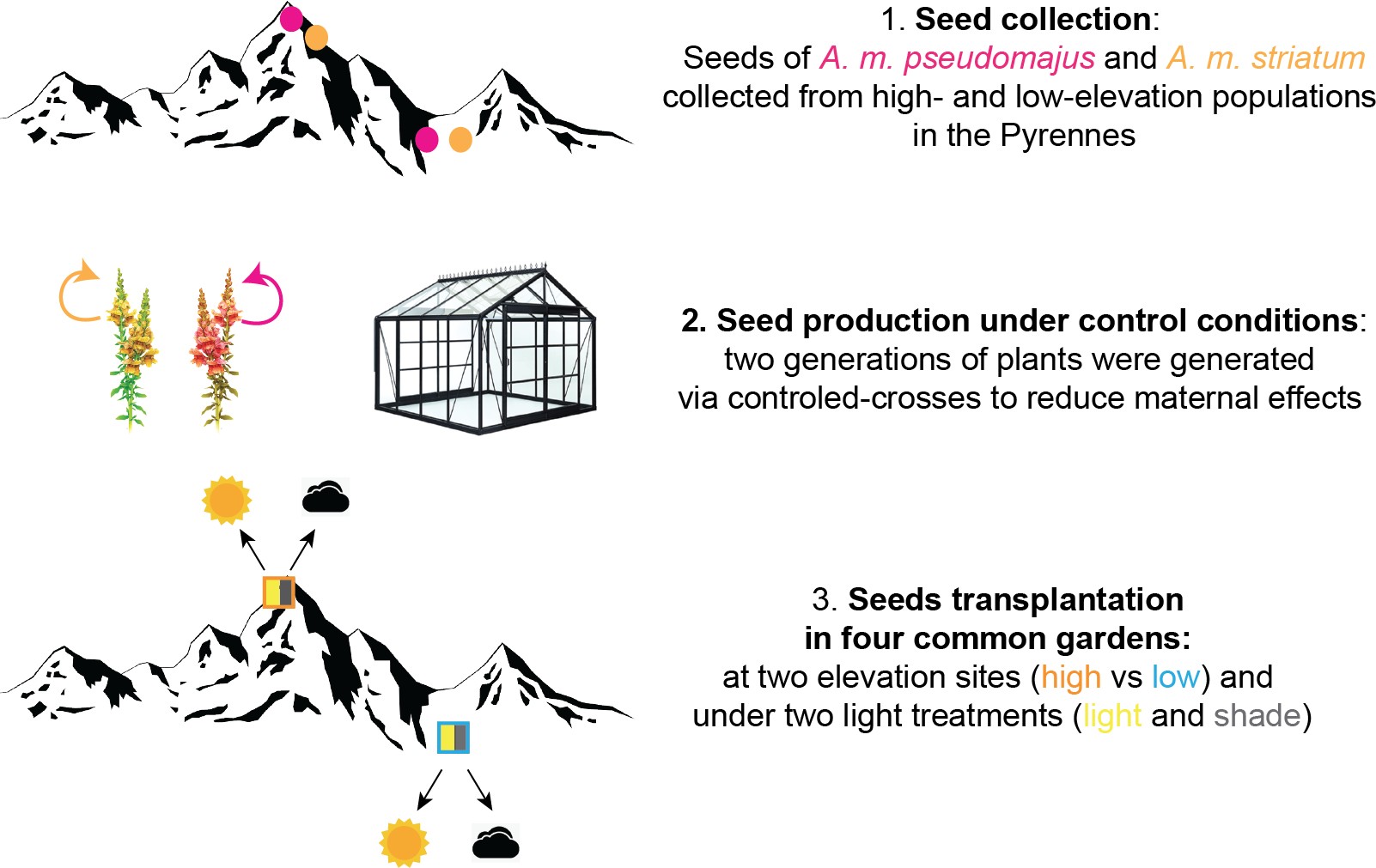


Figure S2: diagram representing the experimental design.

In each wild population, seeds were sampled in October 2011 and randomly collected from mature plants. Seeds sampled in the wild were sown in spring 2012 in individual pots (9 × 9 × 10 cm) filled with universal compost in a greenhouse at the CNRS Experimental Ecology Station in Moulis, France. This first generation of plants germinated and were grown with no nutrient addition under an average temperature from 15 to 28°C and weekly watering. Mature plants were hand-pollinated during the summer 2012 (flowers were bagged before opening to prevent unwanted pollination). Crosses were conducted within populations where mates were assigned randomly. The seeds produced by these crosses constitute the 2012 collection of seed families. Many seeds from each seed family were used in other experiments. We therefore sowed the remaining seeds of each seed family in spring 2014 in a hoop house at ENSFEA (Toulouse, France) where they were watered twice a week before seedlings were transferred outside. This second generation of plants were germinated and grown in individual pots (9 × 9 × 10 cm) filled with universal compost, with no nutrient addition, under temperate climatic conditions (average month temperatures ranging from 20.6 to 21.5°C and cumulative monthly rainfall ranging from 28.3 to 73.4 mm). Plants were supplied with water in case of prolonged drought. Mature plants were hand-pollinated during summer 2014 (flowers were bagged before opening to prevent unwanted pollination). Crosses were conducted within populations where mates were assigned randomly. The seeds resulting from these crosses constitute the 2014 collection of seed families that were used in this study to conduct the 2015 experiments presented here.


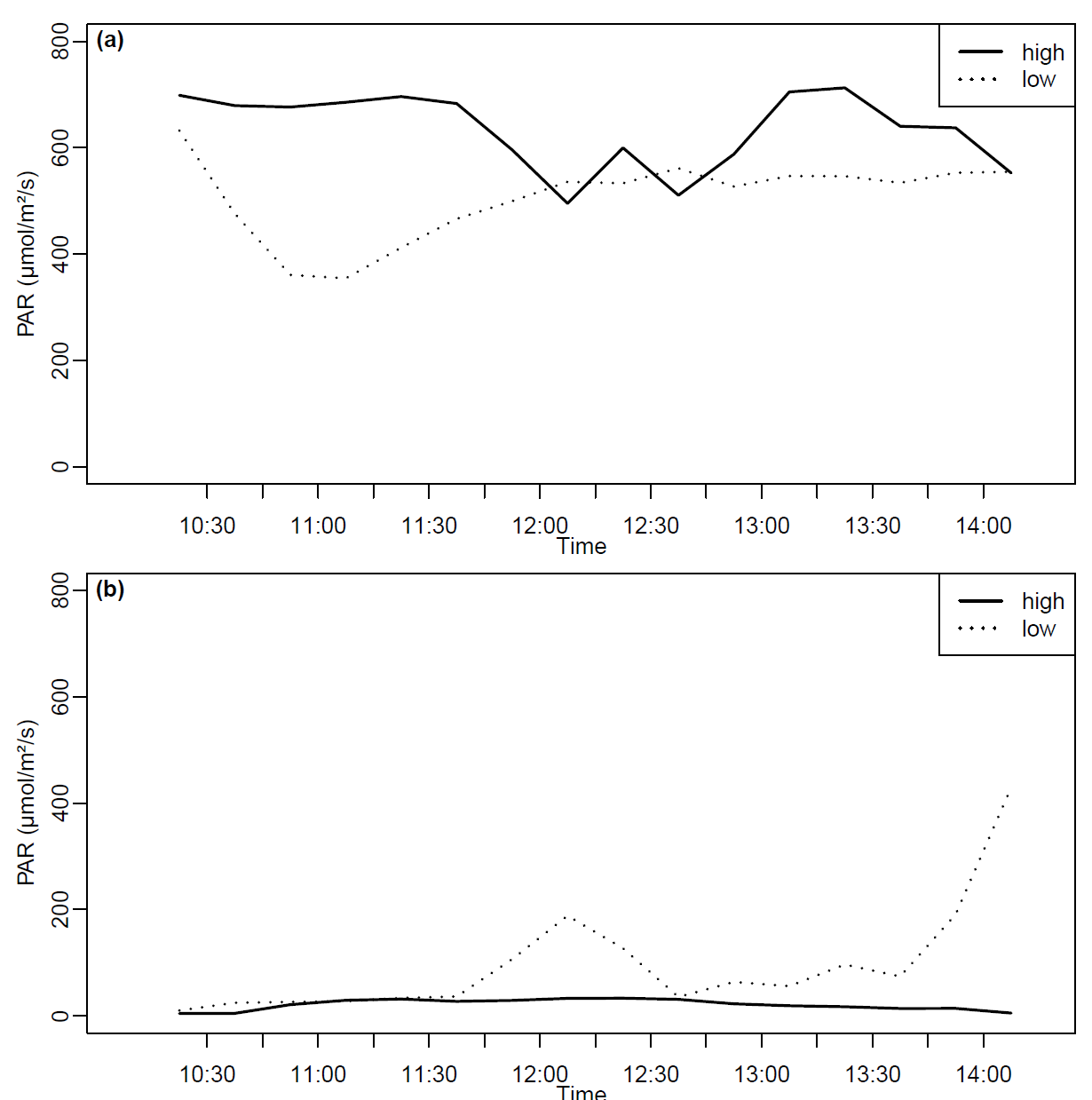


Figure S3: Difference in photosynthetically active radiation (PAR) between high and low elevation sites in a) full light, and b) under the shade of understorey vegetation.

Measurement were acquired by using a fiber-optic spectrometer (AvaSpec-2048; Avantes, Netherlands) with a 25° sensor’s field of view, and a full sky irradiance remote cosine corrector. Photon flux densities of PAR were measured at 60 sec intervals every 15 days from 10 am to 2 pm during the experiment.


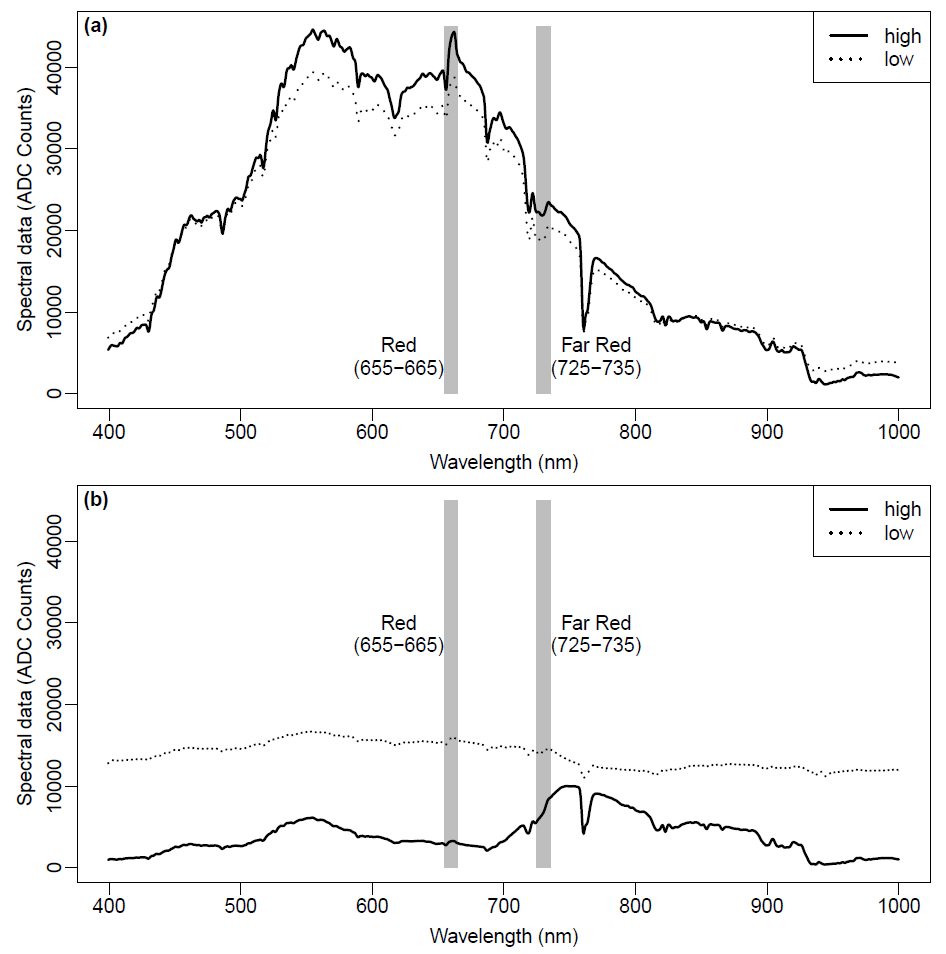


Figure S4: Differences in spectral data between high and low-elevation sites in a) full light, and b) under the shade of understorey vegetation.


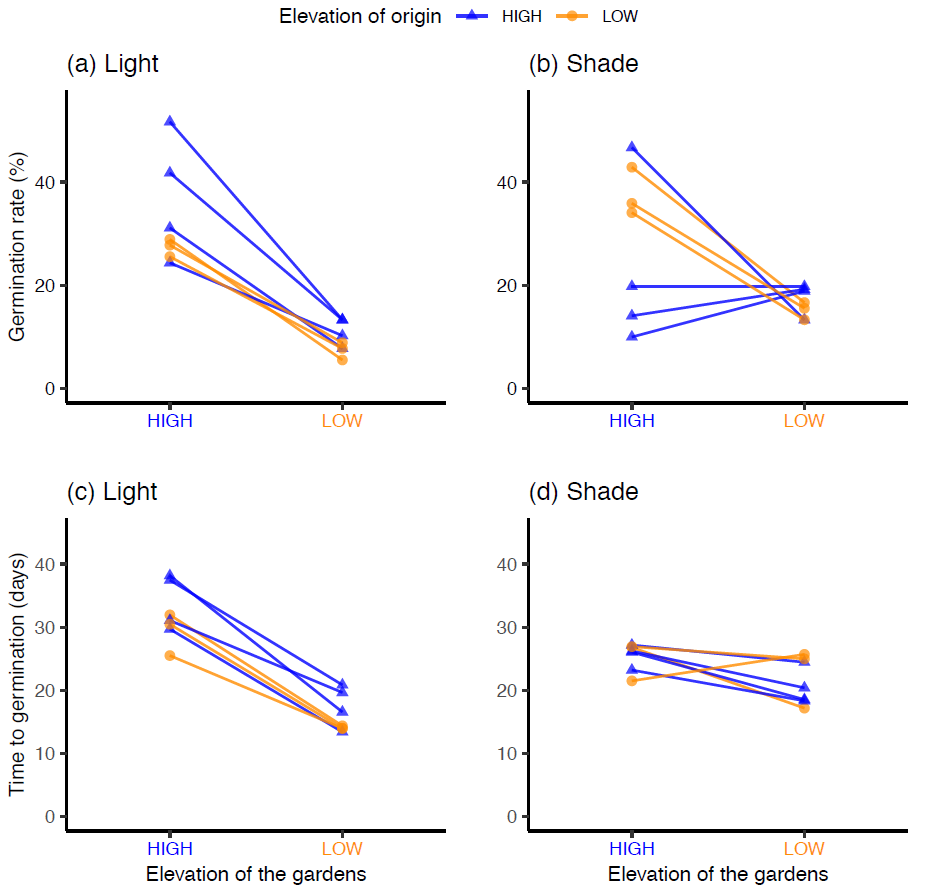


Figure S5: Reaction norms of germination-related traits for seven populations of *Anthirrinum majus striatum* in the two sites (low and high elevations) and under two treatments (full light habitat and understorey shade).

Plots a) and b) refer to the germination rate, c) and d) to the time to germination, a) and c) refer to the full light treatment, b) and d) to the shade treatment. Significant differences are indicated by asterisks. ***: p.value ≤ 0.001 , **: 0.001 < p.value ≤ 0.01, *: 0.01 < p.value ≤ 0.05, “.”: 0.5 < p.value < 0.1, ns“ns”: p.value ≥ 0.1.


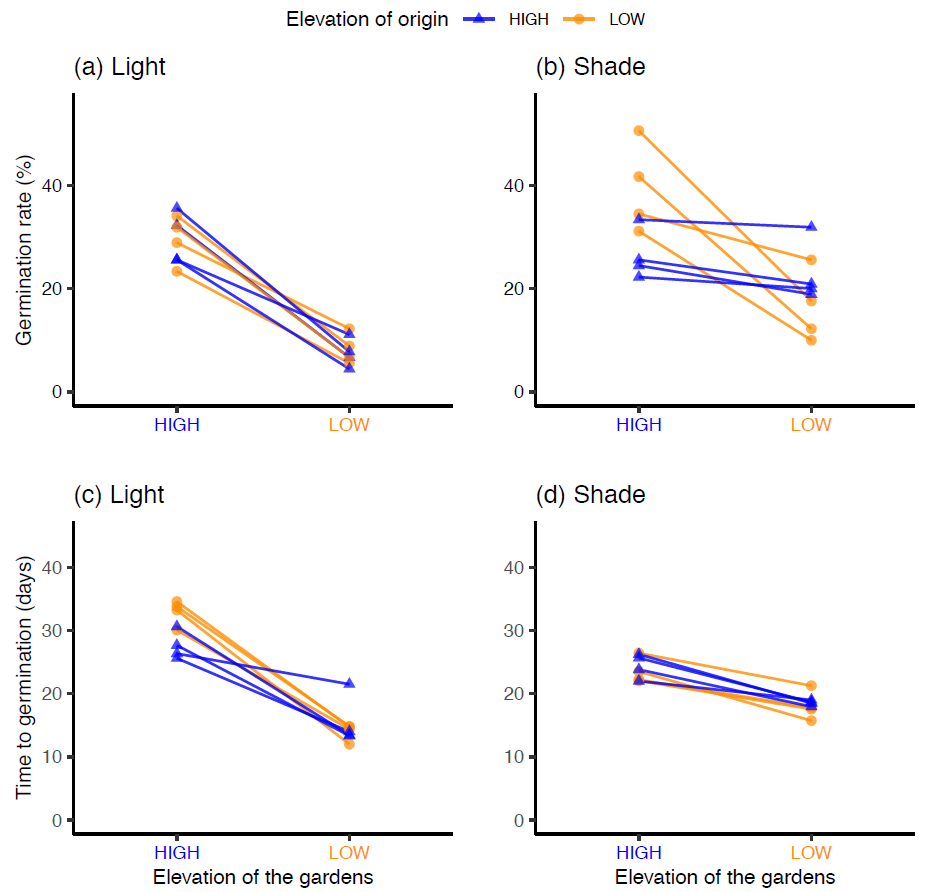


Figure S6: Reaction norms of germination-related traits for eight populations of *Anthirrinum majus pseudomajus* in the two sites (low and high elevations) and under two treatments (full light habitat and understorey shade).

Plots a) and b) refer to the germination rate, c) and d) to the time to germination, a) and c) refer to the full light treatment, b) and d) to the shade treatment. Significant differences are indicated by asterisks. ***: p.value ≤ 0.001 , **: 0.001 < p.value ≤ 0.01, *: 0.01 < p.value ≤ 0.05, “.”: 0.5 < p.value < 0.1, “ns”: p.value ≥ 0.1.
